# Complete Genome Sequence of Acinetobacter indicus Type Strain SGAir0564 Isolated from Tropical Air Collected in Singapore

**DOI:** 10.1128/genomeA.00230-18

**Published:** 2018-05-03

**Authors:** Vineeth Kodengil Vettath, Ana Carolina M. Junqueira, Akira Uchida, Rikky W. Purbojati, James N. I. Houghton, Caroline Chénard, Daniela I. Drautz-Moses, Anthony Wong, Sandra Kolundžija, Megan E. Clare, Kavita K. Kushwaha, Deepa Panicker, Alexander Putra, Nicolas E. Gaultier, Cassie E. Heinle, Balakrishnan N. V. Premkrishnan, Stephan C. Schuster

**Affiliations:** a Singapore Centre for Environmental Life Sciences Engineering, Nanyang Technological University, Singapore; b Asian School of the Environment, Nanyang Technological University, Singapore

## Abstract

Acinetobacter indicus (Gammaproteobacteria) is a strict aerobic nonmotile bacterium. The strain SGAir0564 was isolated from air samples collected in Singapore. The complete genome is 3.1 Mb and was assembled using a combination of short and long reads. The genome contains 2,808 protein-coding genes, 80 tRNAs, and 21 rRNA subunits.

## GENOME ANNOUNCEMENT

Acinetobacter indicus is a Gram-negative, oxidase-negative, catalase-positive, strictly aerobic, nonmotile bacterium (1) classified in the class Gammaproteobacteria. A. indicus was first reported to be isolated from soil samples collected from a hexachlorocyclohexane dump site (1). Subsequently, Acinetobacter indicus-like isolates were recovered from a human clinical sample and presented carbapenem antibiotic resistance (2). Currently, many species of the genus Acinetobacter have been reported from a wide range of environmental sources, like soil, water, food, and areas polluted with hydrocarbons, and even from hosts, such as humans, other vertebrates, and insects (3–5).

A. indicus strain SGAir0564 was isolated from an air sampling study performed in Singapore (global position system [GPS] coordinates 1.345N, 103.679E) using the Andersen single-stage impactor (SKC BioStage). Air was drawn through the impactor using a high-flow pump, where particles were collected onto Trypticase soy agar (TSA; Becton, Dickinson). For isolation, colonies were grown overnight in Luria-Bertani (LB) broth and cultured on TSA at 30°C. Genomic DNA of A. indicus strain SGAir0564 was extracted from these cultures and purified using the Wizard genomic DNA purification kit (Promega). To achieve a high-quality complete genome assembly, both long- and short-read sequencing technologies were utilized. Library preparation was performed with the SMRTbell template prep kit 1.0 (Pacific Biosciences), followed by single-molecule real-time (SMRT) sequencing on the Pacific Sciences RSII platform. Additionally, 300-bp paired-end reads were generated on a MiSeq (Illumina) platform from whole-genome shotgun libraries using the TruSeq Nano DNA library preparation kit.

*De novo* assembly was performed using 55,294 subreads as input with the Hierarchical Genome Assembly Process (HGAP) version 3 (6) implemented in the PacBio SMRT Analysis 2.3.0 package. This step was followed by polishing with Quiver (6) and error correction with Pilon version 1.16 (7), utilizing 793,039 MiSeq paired-end reads. The final consensus assembly generated one contig, containing the complete circular genome of 3,157,380 bp, with a mean G+C content of 45.5% and average coverage of 201.5-fold.

Taxonomic identification using Phyla-AMPHORA (8) showed 99.3% identity to the genus Acinetobacter. In addition, species assignment performed with Microbial Species Identifier (MiSI) (9), based on the genome-wide average nucleotide identity (gANI) metric, showed 97.13% identity to A. indicus strain CIP 110367.

Genome annotation was performed using NCBI’s Prokaryotic Genome Annotation Pipeline (PGAP) version 4.2 (10). PGAP predicted 3,054 genes, including 2,808 protein-coding genes (PCGs), 21 rRNA subunits (seven clusters of 5S, 16S, and 23S), 80 tRNAs, 4 noncoding RNAs, and 141 pseudogenes. Additionally, subsystems-based functional annotation was performed using Rapid Annotations using Subsystems Technology (RAST) (11–13) and assigned most genes to amino acid and derivative metabolism (316) and cofactor, vitamin, prosthetic group, and pigment (235) subsystems.

### Accession number(s).

The complete genome sequence of Acinetobacter indicus SGAir0564 has been deposited in DDBJ/EMBL/GenBank under the accession number CP024620.
