# Correction for Vettath et al., “Complete Genome Sequence of Acinetobacter indicus Strain SGAir0564 Isolated from Tropical Air Collected in Singapore”

**DOI:** 10.1128/MRA.00738-19

**Published:** 2019-08-08

**Authors:** Vineeth Kodengil Vettath, Ana Carolina M. Junqueira, Akira Uchida, Rikky W. Purbojati, James N. I. Houghton, Caroline Chénard, Daniela I. Drautz-Moses, Anthony Wong, Sandra Kolundžija, Megan E. Clare, Kavita K. Kushwaha, Deepa Panicker, Alexander Putra, Nicolas E. Gaultier, Cassie E. Heinle, Balakrishnan N. V. Premkrishnan, Stephan C. Schuster

**Affiliations:** a Singapore Centre for Environmental Life Sciences Engineering, Nanyang Technological University, Singapore; b Asian School of the Environment, Nanyang Technological University, Singapore

## AUTHOR CORRECTION

Volume 6, no. 18, e00230-18, 2018, https://doi.org/10.1128/genomeA.00230-18. Page 1: The title should appear as given in this correction.

